# A Hybrid Localization Algorithm for an Adaptive Strategy-Based Distance Vector-Hop and Improved Sparrow Search for Wireless Sensor Networks

**DOI:** 10.3390/s23208426

**Published:** 2023-10-12

**Authors:** Zhiwei Sun, Hua Wu, Yang Liu, Suyu Zhou, Xiangmin Guan

**Affiliations:** 1 School of Information Science & Electrical Engineering, Shandong Jiaotong University, Jinan 250357, China; 21208038@stu.sdjtu.edu.cn (Z.S.); 22208031@stu.sdjtu.edu.cn (S.Z.); 2 School of General Aviation, Civil Aviation Management Institute of China, Beijing 100082, China; guanxiangmin@camic.cn

**Keywords:** wireless sensor network, localization algorithm, DV-Hop, improve sparrow search

## Abstract

Wireless sensor networks (WSNs) are applied in many fields, among which node localization is one of the most important parts. The Distance Vector-Hop (DV-Hop) algorithm is the most widely used range-free localization algorithm, but its localization accuracy is not high enough. In this paper, to solve this problem, a hybrid localization algorithm for an adaptive strategy-based distance vector-hop and improved sparrow search is proposed (HADSS). First, an adaptive hop count strategy is designed to refine the hop count between all sensor nodes, using a hop count correction factor for secondary correction. Compared with the simple method of using multiple communication radii, this mechanism can refine the hop counts between nodes and reduce the error, as well as the communication overhead. Second, the average hop distance of the anchor nodes is calculated using the mean square error criterion. Then, the average hop distance obtained from the unknown nodes is corrected according to a combination of the anchor node trust degree and the weighting method. Compared with the single weighting method, both the global information about the network and the local information about each anchor node are taken into account, which reduces the average hop distance errors. Simulation experiments are conducted to verify the localization performance of the proposed HADSS algorithm by considering the normalized localization error. The simulation results show that the accuracy of the proposed HADSS algorithm is much higher than that of five existing methods.

## 1. Introduction

Recently, wireless sensor networks (WSNs) have been widely used to realize real-time sensing, accurate identification, and the management of physical worlds [1]. WSNs are composed of many low-power and energy-limited inexpensive sensor nodes that collaborate to sense, collect, process, and transmit information in the coverage area and then send it to the base station. Currently, WSNs are widely used in many fields such as smart cities, national defense, environmental monitoring, and smart homes [2,3,4]. For these applications, node location is indispensable. It would be meaningless if the exact node location cannot be obtained [5].

Existing node localization techniques in WSNs can be classified as range-based or range-free, according to whether a distance measurement between nodes is required [6]. For range-based localization algorithms, additional hardware is needed to measure the distances or angles between nodes whose accuracy is much higher [7]. Right now, a variety of neural network-based technologies are incorporated to improve range-based localization algorithms. However, the accuracy is decreased significantly in complex environments because of interference and high energy consumption [8]. Typical range-based algorithms mainly include time of arrival (ToA) [9], time difference of arrival (TDoA) [10], angle of arrival (AoA) [11], and received signal strength indication (RSSI) [12]. However, for range-free localization algorithms, only network information is needed to realize the node localization, which takes advantage of easy implementation and low energy consumption. Its localization accuracy is generally lower than that of ranging-based algorithms. The DV-Hop algorithm [13], centroid algorithm [14], amorphous algorithm [15], and APIT [16] are all range-free localization algorithms.

The DV-Hop algorithm is a classical range-free localization algorithm that extends the information of a small number of GPS-equipped nodes to the whole network in a hop-by-hop manner to achieve the localization of all nodes without any additional hardware devices. As a popular research topic, many improved DV-Hop algorithms have been proposed. Chen et al. used the average hop distance of a network as a whole instead of the average hop distance obtained with an unknown node from the nearest anchor node and used a two-dimensional hyperbolic algorithm instead of the maximum likelihood estimation method to achieve good results and improve localization accuracy [17]. Zhang et al. analyzed the effect of node collinearity on the localization accuracy of the DV-Hop algorithm and used Voronoi diagrams to zone a wireless sensor network [18]. Peng et al. improved the DV-Hop algorithm using a genetic algorithm and used the bounding box idea to limit the initial population range of the genetic algorithm, which not only accelerated the convergence speed of the algorithm but also improved the localization accuracy [19]. Cui et al. proposed a method using Lévy and Cauchy’s strategy to optimize the cuckoo search algorithm of the DV-Hop-improvement algorithm, and their simulation results showed that the improved algorithm had higher localization performance compared with the DV-Hop algorithm [20]. Santar Pal Singh et al. proposed a PSO-improved DV-Hop algorithm that fully considers the number of hops between nodes when setting the fitness function [21]. Huang et al. proposed a new error-reduction method combining the Manhattan distance with the Euclidean distance to obtain a new hopping frequency and hopping distance and then used a multi-objective genetic algorithm to optimize the unknown node coordinates, which greatly improved the positioning accuracy of the DV-Hop algorithm [22].

Actually, many complex problems are difficult to solve in a reasonable time using the mentioned traditional deterministic optimization algorithms. Many metaheuristic algorithms have been widely adopted in WSN localization due to their flexibility and efficiency. These typical algorithms include the simulated annealing algorithm (SAA) [23], the particle swarm algorithm (PSO) [24], the gray wolf optimization algorithm (GWO) [25], and the whale optimization algorithm (WOA) [26]. Although these algorithms are widely used, they are not perfect. The SSA has a high requirement for the initial temperature of annealing and a slow convergence rate. The PSO converges faster, but it also easily falls into local optima. To solve these problems, selecting appropriate intelligent optimization algorithms becomes important. The sparrow search algorithm is another kind of metaheuristic algorithm that was proposed based on the behavioral activities of sparrow populations [27]. Compared with other algorithms, the sparrow search algorithm takes advantage of fast convergence speed and high convergence accuracy in solving location optimization problems in WSNs.

In this paper, a hybrid localization algorithm for an adaptive strategy-based distance vector-hop and improved sparrow search (HADSS) is proposed. The contributions of this paper are as follows:

1. Since the simple use of multiple communication radii would lead to excessive communication overhead, this paper uses an adaptive hop count strategy to refine the hop counts and a hop count correction factor to refine the hop counts again, which reduces both the hop count error and the communication overhead.
2. A combination of the anchor node trust degree and the weighting method is used to obtain the average hop distance, which takes into account both the global and local information of the anchor nodes. As a result, the average hop distance error can be effectively reduced.
3. An improved sparrow search algorithm is proposed to calculate the coordinates of unknown nodes and reduce the error of coordinate calculation.

The remainder of this paper is organized as follows. The principles of the DV-Hop algorithm and the sparrow search mechanism are provided in Section 2. Improvements to the DV-Hop algorithm and the sparrow search mechanism are given in Section 3. Section 4 presents the implementation process of our proposed algorithm. Algorithm performance metrics and experimental simulation results are shown in Section 5. Finally, this paper is concluded in Section 6.

## 2. Related Work

### 2.1. DV-Hop Algorithm

Phase 1: Obtain the minimum hop count between nodes. First, each anchor node broadcasts a packet containing its location and the hop count field with an initial value of 0. During the broadcasting of packets, the hop count value of packets is increased by 1 with every hop. This means that when receiving this packet, each node records the location of the anchor node and initializes the hop count information. Finally, all nodes in the network record their minimum hop count value with each anchor node and the location coordinates of each anchor node [28].

Phase 2: Calculate the average hop distance between anchor nodes and the distance between unknown nodes and anchor nodes. According to the coordinate information of the anchor node, the straight-line distance between any two anchor nodes is calculated. In the first stage, the minimum hop count value between any two anchor nodes is obtained in phase 1. Then, the equation for average hop distance can be expressed by Equation (1).

(1) $$Hopsize_{i}=\frac{\sum_{j\neq i}^{n}\sqrt{{\left(x_{i}-x_{j}\right)}^{2}+{\left(y_{i}-y_{j}\right)}^{2}}}{\sum_{j\neq i}^{n}h_{ij}}$$

where $Hopsize_{i}$ represents the average hop distance of anchor node i, n represents the number of anchor nodes, $\left(x_{i},y_{i}\right)$ represents the coordinates of anchor node i, $\left(x_{j},y_{j}\right)$ represents the coordinates of anchor node j and $h_{ij}$ represents the minimum hop count between anchor nodes i and j. The unknown node k obtains the average hop distance from the nearest anchor node to itself, and then multiplies it with the minimum hop count obtained in phase 1 to obtain the estimated distance between two nodes. The calculation equation is expressed by Equation (2).

(2) $$d_{ik}=Hopsize_{i}\times h_{ik}$$

where, $d_{ik}$ represents the distance between the unknown node k and the anchor node i, and $h_{ik}$ represents the minimum hop count between the unknown node k and the anchor node i.

Phase 3: Calculate the coordinates of the unknown nodes. In the previous phases, the unknown nodes obtain the estimated distance from all anchor nodes, and the coordinates of the unknown nodes can be calculated using the least squares method. The calculation process is represented by the following equation.

Without considering the error, the distance relationship between the unknown nodes and the anchor nodes can be expressed by Equation (3).

(3) $$\left\{\begin{array}{c} {\left(x_{1}-x_{k}\right)}^{2}+{\left(y_{1}-y_{k}\right)}^{2}=d_{1k}^{2}\\ {\left(x_{2}-x_{k}\right)}^{2}+{\left(y_{2}-y_{k}\right)}^{2}=d_{2k}^{2}\\ \vdots \\ {\left(x_{n}-x_{k}\right)}^{2}+{\left(y_{n}-y_{k}\right)}^{2}=d_{nk}^{2} \end{array}\right.$$

where, $\left(x_{1},y_{1}\right)$, $\left(x_{2},y_{2}\right)$, … $\left(x_{n},y_{n}\right)$ represent the coordinates of *n* anchor nodes and $\left(x_{k},y_{k}\right)$ represents the coordinates of the unknown node k. Subtract the first n−1 equations of Equation (3) from the nth equation to obtain Equation (4).

(4) $$\left\{\begin{array}{c} d_{k1}^{2}-d_{kn}^{2}+x_{n}^{2}-x_{1}^{2}+y_{n}^{2}-y_{1}^{2}=2\left(x_{n}-x_{1}\right)x_{k}+2\left(y_{n}-y_{1}\right)y_{k}\\ d_{k2}^{2}-d_{kn}^{2}+x_{n}^{2}-x_{2}^{2}+y_{n}^{2}-y_{2}^{2}=2\left(x_{n}-x_{2}\right)x_{k}+2\left(y_{n}-y_{2}\right)y_{k}\\ \vdots \\ d_{kn-1}^{2}-d_{kn}^{2}+x_{n}^{2}-x_{n-1}^{2}+y_{n}^{2}-y_{n-1}^{2}=2\left(x_{n}-x_{n-1}\right)x_{k}+2\left(y_{n}-y_{n-1}\right)y_{k} \end{array}\right.$$

Rewrite Equation (4) in the form of $AX_{k}=B$. A, $X_{k}$, and B are shown in Equations (5)–(7).

(5) $$A=2\left[\begin{array}{c} \left(x_{1}-x_{n}\right)\\ \left(x_{2}-x_{n}\right)\\ \vdots \\ \left(x_{n-1}-x_{n}\right) \end{array}\right.\;\;\;\;\;\;\left.\begin{array}{c} \left(y_{1}-y_{n}\right)\\ \left(y_{2}-y_{n}\right)\\ \vdots \\ \left(y_{n-1}-y_{n}\right) \end{array}\right]$$

(6) $$X_{k}=\left[\begin{array}{l} x_{k}\\ y_{k} \end{array}\right]$$

(7) $$B=\left[\begin{array}{c} d_{k1}^{2}-d_{kn}^{2}+x_{n}^{2}-x_{1}^{2}+y_{n}^{2}-y_{1}^{2}\\ d_{k2}^{2}-d_{kn}^{2}+x_{n}^{2}-x_{2}^{2}+y_{n}^{2}-y_{2}^{2}\\ \vdots \\ d_{kn-1}^{2}-d_{kn}^{2}+x_{n}^{2}-x_{n-1}^{2}+y_{n}^{2}-y_{n-1}^{2} \end{array}\right]$$

The coordinates of the unknown nodes can be expressed by Equation (8).

(8) $$X={\left(A^{T}A\right)}^{-1}A^{T}B$$

### 2.2. DV-Hop Error Analysis

1. Minimum Hop Count Error

**Figure 1:**
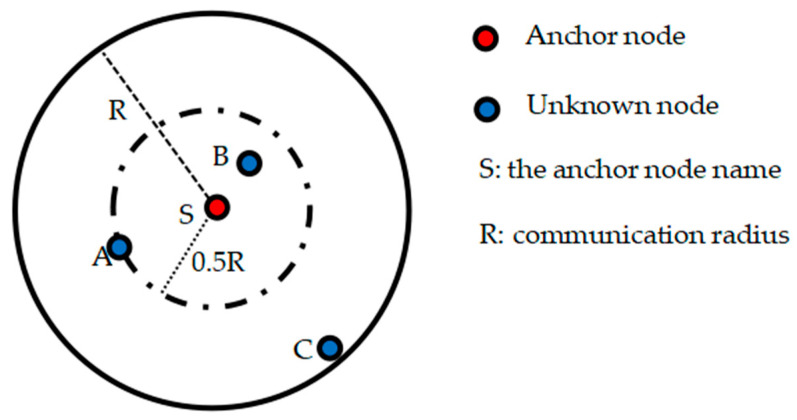
Minimum hop count error diagram.

To solve the coordinates of unknown nodes, the DV-Hop algorithm relies on the hop count and distance information between unknown nodes and anchor nodes. When calculating the minimum hop count, all other nodes within the communication range of the node will record the obtained hop value as 1, but the distance between nodes within the communication radius may have a large gap. As shown in Figure 1, *S* is the anchor node and *A*, *B*, and *C* are the unknown nodes. If the hop count of all three unknown nodes in the communication radius of the anchor node *S* is recorded as 1, then it is equivalent to treating the distances between the unknown nodes *A*, *B*, and *C* and the anchor node *S* as equal. However, it is obvious from the figure that the distances between the unknown nodes *A*, *B*, and *C* and the anchor node *S* are significantly different, which will significantly reduce the localization accuracy.

2. Error in calculating the average hop distance

**Figure 2:**
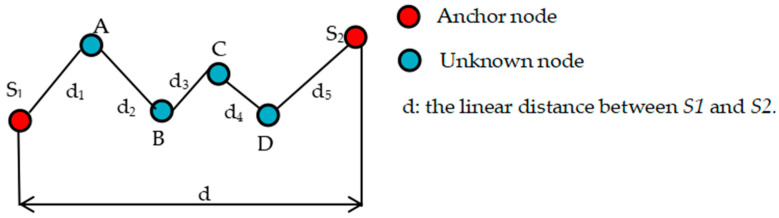
Average hop distance error diagram.

The unknown nodes will obtain the average hop distance from the nearest anchor node, and then calculate the distance between it and the anchor node using Equation (2). In other words, the estimation result of the average hop distance will have a great impact on the localization accuracy. In calculating the average hop distance, the average hop distance is obtained by dividing the linear distance between anchor nodes by the minimum hop count. If the sensors are randomly distributed in a certain area, the paths between the nodes may be zigzag, and the communication between the nodes requires multiple hops to complete. As shown in Figure 2, when the distance between anchor node *S1* and anchor node *S2* is *d* and the minimum hop count is 5, the estimated average hop distance between anchor nodes *S1* and *S2* is d/5,while the actual average hop distance between anchor nodes *S1* and *S2* is $\left(d_{1}+d_{2}+d_{3}+d_{4}+d_{5}\right)/5$. Because $d<\left(d_{1}+d_{2}+d_{3}+d_{4}+d_{5}\right)$, there is an error in the average hop distance estimated using the DV-Hop algorithm compared with the actual average hop distance. The unknown node obtains the average hop distance from the nearest anchor node. Once the average hop distance error of the anchor node itself are large, then the unknown node will have accumulated errors when calculating its coordinates, which will largely reduce the localization accuracy.

3. Error in calculating coordinates

When DV-Hop calculates the coordinates of the unknown nodes, the least squares method is generally used. However, the reference equation chosen by the least squares method has a great influence on the positioning results, especially if the chosen equation corresponds to a large hop count between the anchor node and the unknown node, which will cause accumulated errors.

### 2.3. Sparrow Search

The sparrow search algorithm is a new swarm intelligence optimization algorithm that was proposed in 2020. According to the different tasks that sparrows undertake in a population, the sparrow population is divided into discoverers, followers, and scouts.

1. The discoverer takes on the task of finding food in the sparrow population and plays a dominant role in the population. The location renewal mechanism of discoverers is shown in Equation (9).

(9) $$X_{i,j}^{t+1}=\left\{\begin{array}{ll} X_{i,j}^{t}\cdot \exp\left(\frac{-i}{\alpha \cdot M}\right) & if\;R_{2}<ST\\ X_{i,j}^{t}+Q\cdot L & if\;R_{2}\geq ST \end{array}\right.$$

where, $X_{i,j}^{t+1}$ and $X_{i,j}^{t}$ represent the position of the ith sparrow in the jth dimension at t+1 and t iterations, respectively, M represent the maximum number of iterations, α is a random number on $\left[0,1\right]$, Q is a random number obeying a normal distribution, and L is a 1×D-dimensional matrix with all its elements being 1. $R_{2}$ is the warning value of the population, $R_{2}\in \left[0,1\right]$, and ST is the safety threshold, $ST\in \left[0.5,1\right]$. $R_{2}<ST$ means that the sparrow population is not in danger, whereas $R_{2}\geq ST$ means that the sparrow population is in danger and needs to move to a safe place to feed.

2. The follower will find the discoverer with a high fitness value to update its position, and the position update mechanism of the follower is shown in Equation (10).

(10) $$X_{i,j}^{t+1}=\left\{\begin{array}{ll} Q\cdot \exp\left(\frac{X_{worst}^{t}-X_{i,j}^{t}}{i^{2}}\right) & if\;i>\frac{n}{2}\;\\ X_{P}^{t+1}+\left|X_{i,j}^{t}-X_{P}^{t+1}\right|\cdot A^{+}\cdot L & if\;i\leq \frac{n}{2} \end{array}\right.$$

where $X_{worst}^{t}$ and $X_{P}^{t+1}$ represent the global worst sparrow position and the best sparrow position at the tth and t+1th iteration, respectively. n represents the number of sparrows in the population. $A^{+}$ is a 1×D-dimensional matrix with elements randomly assigned dimension 1 or −1, while $A^{+}=A^{T}{\left(AA^{T}\right)}^{-1}$.

3. Scouts will give an early warning of danger that allows the whole population to escape from it. The scout’s location update mechanism is shown in Equation (11).

(11) $$X_{i,j}^{t+1}=\left\{\begin{array}{ll} X_{best}^{t}+\beta \cdot \left|X_{i,j}^{t}-X_{best}^{t}\right| & if\;f_{i}\neq f_{g}\\ X_{i,j}^{t}+K\left(\frac{\left|X_{i,j}^{t}-X_{worst}^{t}\right|}{\left(f_{i}-f_{\omega }\right)+\varepsilon }\right) & if\;f_{i}=f_{g} \end{array}\right.$$

where $X_{best}^{t}$ is the global optimum position of the tth iteration, β is the step correction coefficient and obeys the standard normal distribution $K=\left[-1,1\right]$, and $f_{i}$, $f_{g}$ and $f_{\omega }$ denote the current sparrow fitness value, the global optimum sparrow fitness value and the global worst sparrow fitness value, respectively. ε is a small constant to avoid the case of a zero denominator.

The sparrow search algorithm has the advantages of a simple framework, few control parameters, good flexibility, and easy implementation, but it still has the problems of low convergence accuracy and difficulty in hopping out of local extremes.

## 3. Model and Methodology

Based on the analysis, we propose strategies for the three phases of the DV-Hop algorithm to improve its deficiencies.

### 3.1. Adaptive Hop Count Strategy

First, to address the problem that the first stage of the DV-Hop algorithm sets the hop count of all nodes within the communication radius of the anchor node to 1, which in turn leads to an error in the minimum hop count, an adaptive hop count strategy is used to refine the minimum hop count of the nodes within the communication radius of the anchor node with different distances from the anchor node to achieve the purpose of reducing the error [29]. The adaptive hop count strategy is shown in Equation (12).

(12) $$m=\left\lceil \left(\frac{n}{N}+\frac{R}{L}\right)*\sigma \right\rceil$$

where m denotes the minimum hop count of nodes within the refined communication radius of the anchor node. Taking Figure 1 as an example, assuming m=2, it means that the anchor node broadcasts to the whole network with a double communication radius. There are then two minimum hop counts within the communication radius of the anchor node, 0.5 and 1, the minimum hop count between node *A* and node *B* and anchor node *S* is 0.5, and the minimum hop count between node *C* and anchor node *S* is 1. n represents the number of anchor nodes, N represents the total number of nodes, R represents the maximum communication distance between nodes, L represents the maximum length of the network coverage area, and σ represents the multiplicity factor. The specific value is set according to the hop subdivision requirement in the network coverage area and the information related to node deployment. ⌈ ⌉ stands for rounding upward.

As can be seen from Equation (12), the value of m is proportional to the ratio of the anchor node and the ratio of the communication radius to the maximum length of the network coverage area. For example, if the number of anchor nodes is increased while other conditions remain unchanged, then the number of nodes within the range of one hop that can communicate directly with the anchor node becomes larger. At the same time, the value of m becomes larger so that the minimum number of hops of nodes within the communication range of the anchor node is more refined and can reduce the error to a greater extent. Conversely, a smaller value of m reduces the unnecessary communication overhead while ensuring the minimum degree of hop subdivision required.

Second, to further minimize the error, the minimum hop count is corrected again using the hop count correction factor [30]. To define the ideal hop count $H_{ij}$, $H_{ij}$ can be expressed by Equation (13).

(13) $$H_{ij}=\frac{d_{ij}}{R}$$

where $d_{ij}$ denotes the distance between anchor nodes i and j. To define the hop count deviation $\gamma_{ij}$, $\gamma_{ij}$ can be expressed by Equation (14).

(14) $$\gamma_{ij}=\frac{h_{ij}-H_{ij}}{h_{ij}}$$

where $h_{ij}$ denotes the hop count between anchor nodes i and j. The magnitude of the value of $\gamma_{ij}$ is an indication of the difference between the estimated and ideal hops, and the larger the $\gamma_{ij}$, the larger the difference between the estimated and ideal hops. To define the correction factor $\omega_{ij}$, $\omega_{ij}$ can be expressed by Equation (15).

(15) $$\omega_{ij}=1-\gamma_{ij}^{2}$$

The corrected hop count can be expressed by Equation (16).

(16) $$h_{ij}^{’}=\omega_{ij}h_{ij}$$

### 3.2. Average Hop Distance Optimization

DV-Hop uses the unbiased estimation criterion, which is given in Equation (1), to calculate the average hop distance between anchor nodes. However, it is found that the errors obey a Gaussian distribution in general [31], so it is more reasonable to use the mean square error criterion to calculate the average hop distance than to use the bias and variance. We use the mean square error criterion to calculate the average hop distance, and the formula of the average hop distance can be expressed by Equation (17).

(17) $$Hopsize_{i}=\frac{\sum_{i\neq j}hop_{ij}\cdot d_{ij}}{\sum_{i\neq j}hop_{ij}^{2}}$$

where $hop_{ij}$ is the modified minimum hop count between anchor nodes and $d_{ij}$ is the distance between anchor nodes, which can be expressed by Equation (18).

(18) $$d_{ij}=\sqrt{{\left(x_{i}-x_{j}\right)}^{2}+{\left(y_{i}-y_{j}\right)}^{2}}$$

In the second phase of DV-Hop algorithm, the unknown node obtains the average hop distance from the anchor node closest to itself, which is too simple to select. The main characteristic of nodes in WSNs is the randomness of their distribution, and it is important to make full use of the information of more anchor nodes to compute the average hop distance of the unknown node. For this reason, a combination of the anchor node trust degree and the weighting is then adopted to correct the average hop distance obtained by unknown nodes.

First, the central idea of the weighting method is to strengthen the influence of anchor nodes in the WSN that are close to the unknown node; the closer the anchor node is to the unknown node, the higher the weight, and vice versa, the lower the weight. The weighting coefficient can be expressed by Equation (19).

(19) $$\varphi_{i}=\frac{\frac{1}{hop_{ij}}}{\sum_{i=1}^{n}\frac{1}{hop_{ij}}}$$

where $\varphi_{i}$ is the weight coefficient and $hop_{ij}$ represents the hop count between the unknown node and the anchor node. The average hop distance of the unknown nodes can be represented by Equation (20).

Secondly, the central idea of the trust degree method is that the anchor nodes add a trust degree, and according to the size of the trust degree coefficient, the influence of the anchor nodes with a large trust degree coefficient is strengthened.

Since the average hop distance calculated by each anchor node is different, two different estimated distances are generated between anchor nodes *S1* and *S2*. In order to balance the errors generated by the estimated distances, the estimated distance $d_{ij}^{’}$ is taken as the average of the two distances.

(20) $$d_{ij}^{’}=\frac{\left(Hopsize_{i}\cdot h_{ij}\right)+\left(Hopsize_{j}\cdot h_{ij}\right)}{2}$$

The actual distance between anchor nodes *S1* and *S2* is $d_{ij}$, and the average per-hop error $e_{ij}$ between *S1* and *S2* can be calculated with the help of the error between $d_{ij}$ and $d_{ij}^{\prime }$.

(21) $$e_{ij}=\frac{\left|d_{ij}^{\prime }-d_{ij}\right|}{h_{ij}}$$

The global average per-hop error $E_{ij}$ of *S1* can be obtained by accumulating the average per-hop errors between *S1* and all other anchor nodes to find the average value.

(22) $$E_{ij}=\frac{\sum_{i\neq j}^{n}e_{ij}}{n-1}$$

The trust factor is defined as the ratio of the inverse of the global average per-hop error of this anchor node to the inverse of the global average per-hop error of all anchor nodes.

(23) $$\xi_{i}=\frac{\frac{1}{E_{i}}}{\sum_{i=1}^{n}\left(\frac{1}{E_{i}}\right)}$$

The average hop distance of unknown nodes can be expressed by Equation (24).

(24) $$dhop_{\xi }=\sum_{i=1}^{n}\xi_{i}\cdot Hopsize_{i}$$

In order to address the fact that a single method cannot fully and accurately reflect the hop distance in the network and reduce the risk of error accumulation, the average hop distances obtained with the two methods are summed and averaged to obtain the final average hop distance $dhop_{fin}$.

(25) $$dhop_{fin}=\frac{dhop_{\varphi }+dhop_{\xi }}{2}$$

### 3.3. Improved Sparrow Search Mechanism

1. Fitness function setting

Based on the distances between the unknown nodes and the anchor nodes obtained in the first two phases of the DV-Hop algorithm, the following set of equations can be obtained, as shown in Equation (26).

(26) $$\left\{\begin{array}{c} {\left(x_{1}-x_{k}\right)}^{2}+{\left(y_{1}-y_{k}\right)}^{2}={\hat{d}}_{k1}^{2}\\ {\left(x_{2}-x_{k}\right)}^{2}+{\left(y_{2}-y_{k}\right)}^{2}={\hat{d}}_{k2}^{2}\\ \vdots \\ {\left(x_{n}-x_{k}\right)}^{2}+{\left(y_{n}-y_{k}\right)}^{2}={\hat{d}}_{kn}^{2} \end{array}\right.$$

where ${\hat{d}}_{ki}^{2}$ is the distance between the unknown node k and the anchor node i estimated in the first two phases of the DV-Hop algorithm, and the error between ${\hat{d}}_{ki}^{2}$ and the actual distance is assumed to be $f_{ki}$. $f_{ki}$ can be expressed by Equation (27).

(27) $$f_{ki}=\left|{\left(x_{i}-x_{k}\right)}^{2}+{\left(y_{i}-y_{k}\right)}^{2}-{\hat{d}}_{ki}^{2}\right|$$

Then, the fitness function can be set as follows:

(28) $$fitness=\sum_{i=1}^{n}\left|{\left(x_{i}-x_{k}\right)}^{2}+{\left(y_{i}-y_{k}\right)}^{2}-{\hat{d}}_{ki}^{2}\right|$$

where $\left(x_{1},y_{1}\right)$, $\left(x_{2},y_{2}\right)$, … $\left(x_{n},y_{n}\right)$ represent the coordinates of *n* anchor nodes and $\left(x_{k},y_{k}\right)$ represents the coordinates of the unknown node k.

2. Good point-set strategy

**Figure 3:**
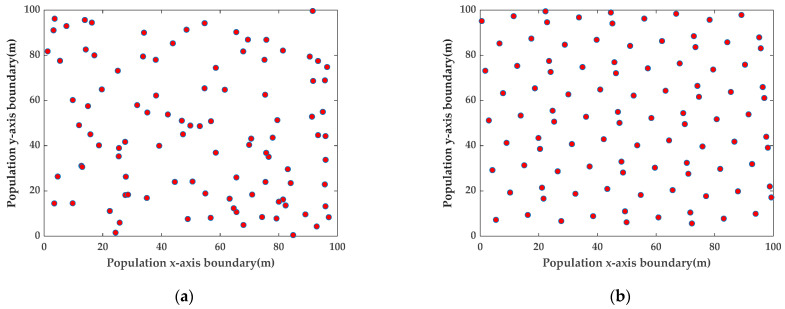
Initial population distribution maps. (**a**) Random strategy to initialize the population distribution map. (**b**) Good point-set strategy to initialize the population distribution map.

The population initialization of the original sparrow search algorithm is random, and the initial population diversity is poor, which will reduce the efficiency of the algorithm for finding the optimum solution. In this paper, we introduce the good point-set strategy to optimize the initial sparrow population, increase the initial population diversity [32], and make the initial population distribution more uniform, as shown in Figure 3. The initial population generated using the good point-set initialization strategy under the same conditions is more uniform than the randomly generated initial population.

3. Improved discoverer position update mechanism

By analyzing Equation (9), it can be concluded that the previous generation discoverer’s position and the discoverer’s position change factor play a decisive role in the discoverer’s position. The value of the position change factor decreases randomly with the increase in the discoverer number, and when the value of the position change factor is larger, the discoverer’s position changes obviously, which enables the discoverer to explore food in a wider area. When the value of the position change factor is smaller, the discoverer’s position also changes less, which enables the discoverer to explore locally near the optimum solution and improves the algorithm’s local search capability. Therefore, the value of the position change factor is especially important for the discoverer’s location update mechanism. In order to better balance the discoverer between global exploration and local exploration, we improve the discoverer position update mechanism [33], and the improved discoverer position update mechanism is shown by Equation (29).

(29) $$X_{i,j}^{t+1}=\left\{\begin{array}{ll} X_{i,j}^{t}\cdot \frac{2}{\exp{\left(\frac{4i}{\alpha \cdot M}\right)}^{2}} & if\;R_{2}<ST\\ X_{i,j}^{t}+Q\cdot L & if\;R_{2}\geq ST \end{array}\right.$$

**Figure 4:**
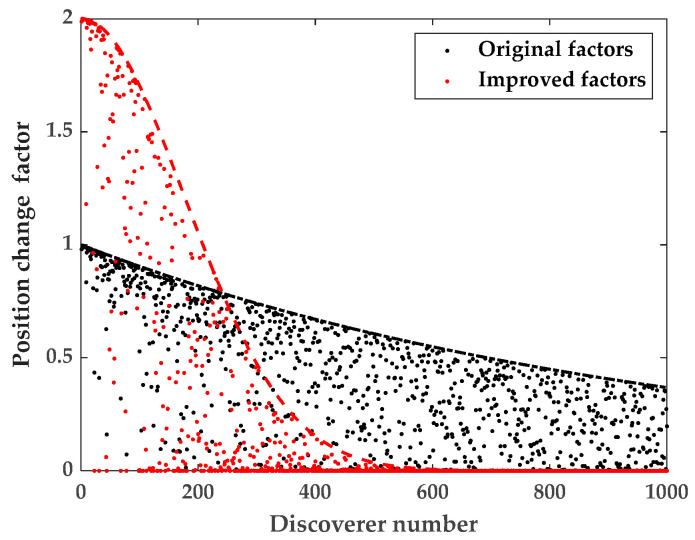
Discoverer search strategy.

Let $a=\exp\left(\frac{-i}{\alpha \cdot M}\right)$ and $b=\frac{2}{\exp\left(\frac{4i}{\alpha \cdot M}\right)}$. According to Figure 4, it can be seen that in the smaller part of the discoverer number, the range of *b* values is larger than *a*, and the discoverer can search for the best at a larger step, and in the larger part of the discoverer number, the range of *b* values is smaller than *a*, and the discoverer can search locally at a smaller step to improve the accuracy of the search.

4. Adaptive *t*-distribution perturbation strategy

The *t*-distribution, also called the student distribution, has a degrees of freedom parameter *m* that determines the shape of the curve of the *t*-distribution [34]. The *t*-distribution probability density function is represented by Equation (30):

(30) $$p\left(x\right)=\frac{\Gamma \left(\frac{m+1}{2}\right)}{\sqrt{m\pi }\times \Gamma \left(\frac{m}{2}\right)}\times {\left(1+\frac{x^{2}}{m}\right)}^{-\frac{m+1}{2}},-\infty <x<+\infty$$

where:

(31) $$\Gamma \left(\frac{m+1}{2}\right)=\int_{0}^{+\infty }x^{\frac{m+1}{2}-1}e^{-x}dx$$

**Figure 5:**
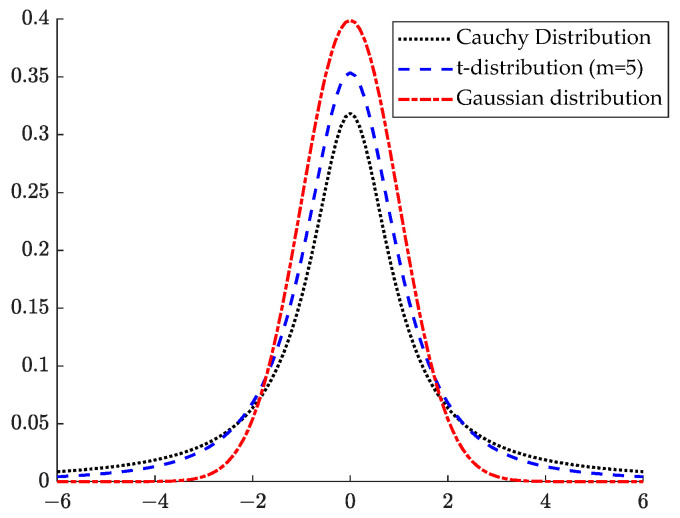
Probability density plots of the Cauchy distribution, *t*-distribution, and Gaussian distribution.

The degree of freedom parameter m=1 is an important parameter that determines the nature and shape of the *t*-distribution. When m=1, the *t*-distribution is the same as the Cauchy distribution, and as m=1 increases, the *t*-distribution gradually approaches the Gaussian distribution until m→∞ and the *t*-distribution becomes the same as the Gaussian distribution. The probability density plots of the *t*-distribution, Cauchy distribution and Gaussian distribution are shown in Figure 5. 

In order to prevent the algorithm from falling into the local optimum in the iterative search process and being unable to hop out, this paper uses the adaptive *t*-distribution perturbation mechanism with the number of iterations iter as the parameter of degrees of freedom to help the algorithm hop out of the local optimum, enhance the global search capability of the algorithm in the early iteration and the local exploration capability in the late iteration, and improve the convergence accuracy of the algorithm. The specific position update mechanism can be expressed by Equation (32).

(32) $$X_{i}^{t+1}=X_{i}^{t}+X_{i}^{t}\cdot t\left(iter\right)$$

where $X_{i}^{t+1}$ is the position of the sparrow after *t*-distribution perturbation, $X_{i}^{t}$ is the position of the ith sparrow in the tth iteration, and $t\left(iter\right)$ is the *t*-distribution variation operator. The perturbation factor is added when the position is updated, and the position information of the previous generation is considered, which helps the algorithm to hop out of the local optimum.

When using the *t*-distribution perturbation mechanism, in order to avoid an excessive increase in the operating cost of the algorithm, this paper uses the adaptive probability *p* to regulate the use of the *t*-distribution perturbation mechanism in the process of finding the optimum. The adaptive probability *p* can be expressed by Equation (33).

(33) $$p=\delta_{1}-\delta_{2}\cdot \frac{\left(M-i\right)}{M}$$

where $\delta_{1}$ represents the maximum value of p and $\delta_{2}$ represents the variation in p. In this paper, we choose $\delta_{1}=0.5$ and $\delta_{2}=0.1$.

## 4. Realization of Proposed Algorithm

The complete implementation process and flowchart of our proposed algorithm are given in this section.

### 4.1. Improvement in DV-Hop Algorithm

1. After initializing the network, the anchor nodes broadcast information to the network using an adaptive hop count strategy according to the network deployment until all nodes obtain the refined minimum hop count, and then the hop count between anchor nodes is corrected again according to the hop count correction factor.
2. The average hop distance between anchor nodes is calculated according to the mean square error criterion. The unknown nodes obtain the average hop distance according to the combination of the anchor node trust degree and the weighting method and multiply it with the obtained minimum hop count to obtain the estimated distance between all unknown nodes and all anchor nodes.
3. The improved sparrow search algorithm is used to locate the unknown nodes instead of the least squares method.

### 4.2. Steps and Flow Chart of the HADSS Algorithm

1. Initialize the WSN settings with a node deployment area of a square area of L×L meters, sensor numbers *N*, anchor node numbers *n,* and communication radius *R*.
2. The anchor nodes adaptively select the number of communication radii and broadcast information to the network based on the initial conditions in the network by the results calculated using Equation (12) until all nodes have obtained the minimum hop count between them and the anchor node, and then makes a secondary correction to the hop count between the anchor nodes.
3. Calculate the average hop distance between anchor nodes. The unknown nodes obtain the average hop distance according to the combination of the anchor node trust degree and the weighting method and then multiply it with the obtained minimum hop count to obtain the estimated distance between all unknown nodes and all anchor nodes.
4. The good point-set strategy is used to initialize the sparrow population with the number of populations *Num*, the maximum number of iterations *M*, the population safety threshold *ST*, and other parameters.
5. Calculate the fitness values of the population according to Equation (28), rank them from smallest to largest, and select the top 20% of the fitness ranking as discoverers and the rest as followers.
6. According to Equations (29), (10), and (11), the positions of discoverers, followers, and scouts are updated, and the population fitness values are calculated.
7. Perturb the updated positions using adaptive *t*-distribution variation according to Equation (32), calculate the fitness values before and after the variation using Equation (28) and compare them, and keep the positions and fitness values of the individuals with the best fitness values.
8. Determine whether the current iteration number *iter* reaches the maximum iteration number *M*. If yes, proceed to the next step; if not, return to step (3).
9. Output the position of the global optimum sparrow.

**Figure 6:**
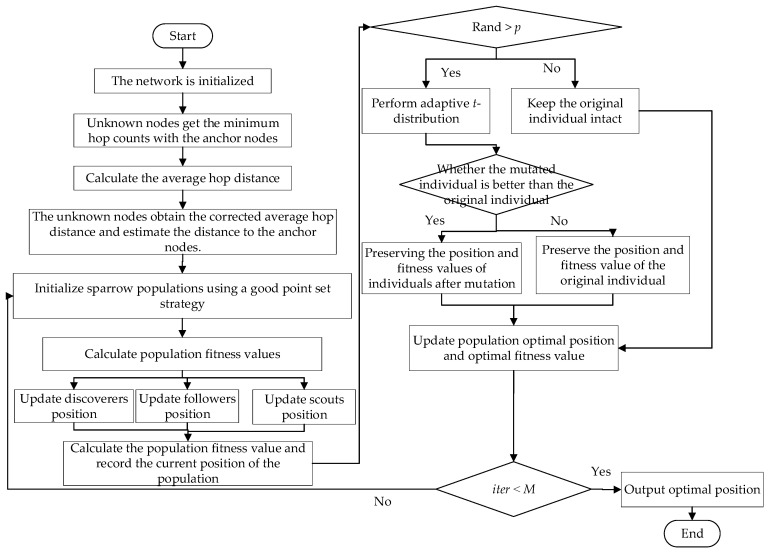
Flow chart of the HADSS algorithm.

A flow chart of the HADSS algorithm is shown in Figure 6.

## 5. Experimental Results and Analysis

Algorithm performance evaluation metrics and experimental simulation results are given in this section.

### 5.1. Scenario Settings

**Figure 7:**
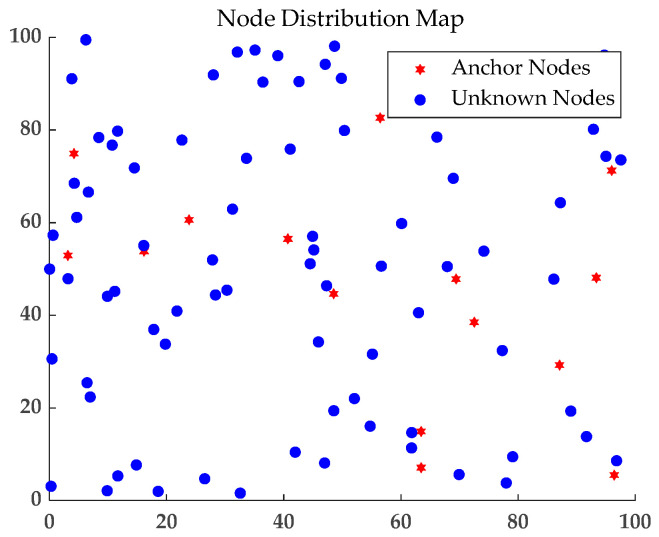
Node distribution map.

In order to prove the superiority of the algorithm proposed in this paper, MATLAB 2021b is used as the experimental simulation platform for simulation experiments. The computer configuration is Windows 10 operating system, Intel Core i5-8265U@1.6GHz processor, 16GB RAM. The simulation parameters are set in Table 1 and Figure 7.

**Figure 8:**
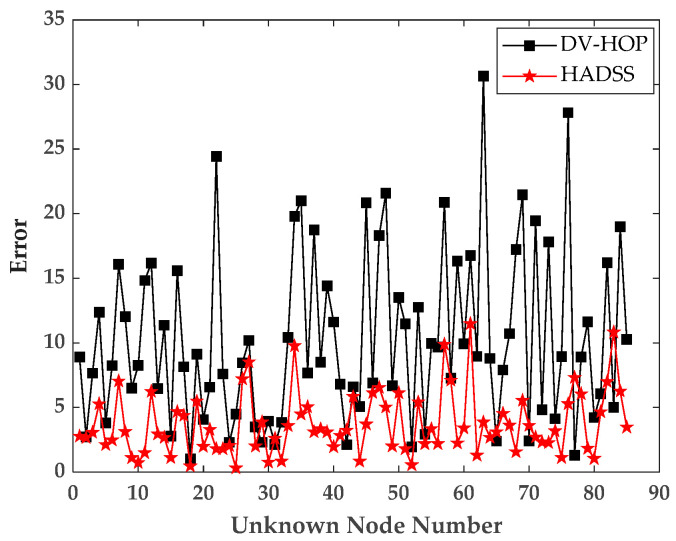
Unknown nodes localization error map.

According to the initial WSN experimental simulation parameters, the simulation experiments were conducted, and the error fold diagram was obtained, as shown in Figure 8.

According to Figure 8, it can be seen that the HADSS algorithm has a smaller localization error compared with the DV-Hop algorithm. In order to obtain more rigorous experimental conclusions, several experiments were conducted in different environments including an anchor node numbers experiment, a communication radius experiment, a sensor numbers experiment, and a scenario sizes experiment. The results were compared with the DV-Hop algorithm, PSODV-Hop algorithm [35], ABCDV-Hop algorithm [36], FADV-Hop algorithm [37], and ISSADV-Hop algorithm [38]. In order to show the experimental results more intuitively and reduce the experimental chance, the normalized average localization error is introduced as the evaluation index of the algorithm, and each experiment is repeated 30 times and the average value is taken. The normalized localization error can be expressed by Equation (34).

(34) $$Error=\frac{\sum_{i=1}^{N-n}\sqrt{{\left(x_{i}-{\hat{x}}_{i}\right)}^{2}+{\left(y_{i}-{\hat{y}}_{i}\right)}^{2}}}{\left(N-n\right)\cdot R}$$

where $\left(x_{i},y_{i}\right)$ denotes the true coordinates of the unknown nodes, $\left({\hat{x}}_{i},{\hat{y}}_{i}\right)$ denotes the estimated coordinates of the unknown nodes, and $\left(N-n\right)$ denotes the unknown node numbers.

### 5.2. Effects of Communication Radius

**Figure 9:**
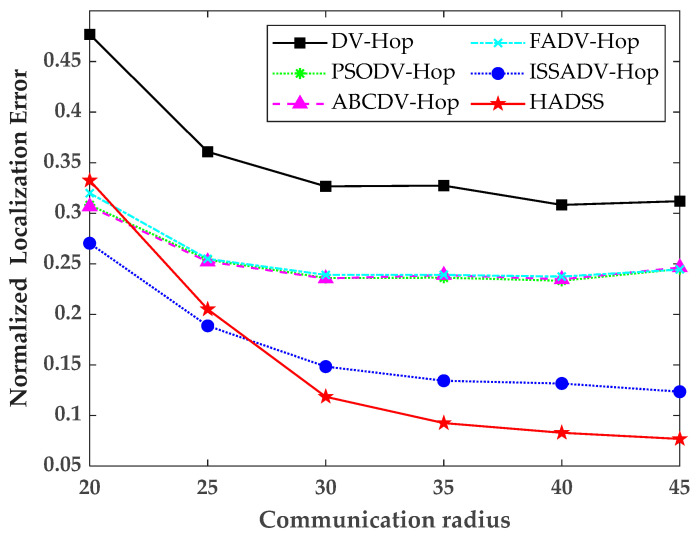
Variation in the normalized localization error for different communication radii.

To study the localization performance of the HADSS algorithm under different communication radii, the communication radius was increased from 20 to 45 m, each time by 5 m, while keeping the anchor node number, the sensor numbers, and the scenario sizes constant. The results of the simulation experiments are shown in Figure 9 and Table 2.

From Figure 9 and Table 2, it can be concluded that with the gradual increase in the communication radius, the normalized localization error of all six algorithms tends to decrease. The average normalized localization error of the DV-Hop algorithm is 0.3519, the average localization error of the PSODV-Hop algorithm is 0.2520, the average normalized localization error of the ABCDV-Hop algorithm is 0.2523, the average normalized localization error of the FADV-Hop algorithm is 0.2557, the average normalized localization error of the ISSADV-Hop algorithm is 0.1661, and the average normalized localization error of the HADSS algorithm is 0.1513. The analysis shows that the average normalized localization error of the HADSS algorithm is 57.00% smaller than that of the DV-Hop algorithm, 39.96% smaller than that of the PSODV-Hop algorithm, 40.01% smaller than that of the ABCDV-Hop algorithm, 40.82% smaller than that of the FADV-Hop algorithm, and 8.91% smaller than that of the ISSADV-Hop algorithm.

### 5.3. Effects of Sensor Numbers

**Figure 10:**
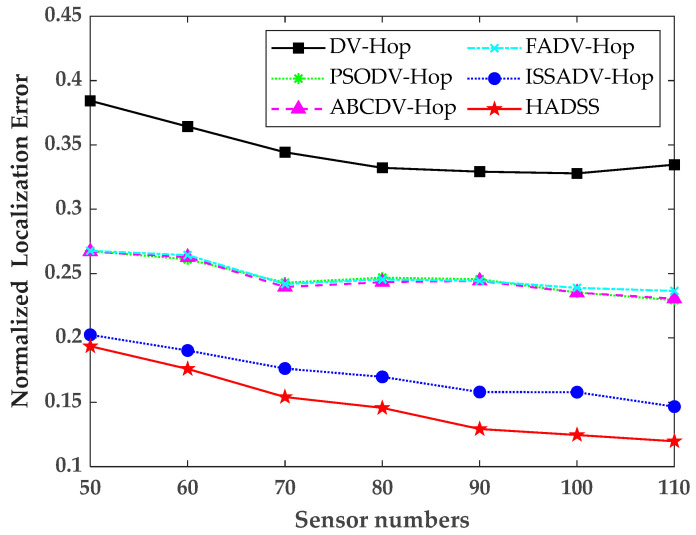
Variation in the normalized localization error for different sensor numbers.

To study the localization performance of the HADSS algorithm with different sensor node numbers, the sensor numbers were increased from 50 to 110, 10 at a time, while keeping the anchor node numbers, communication radius, and scenario sizes constant. The simulation results are shown in Figure 10 and Table 3.

From Figure 10 and Table 3, it can be concluded that with increasing sensor numbers, the normalized localization error of all six algorithms shows a decreasing trend, and the normalized localization error of the HADSS algorithm is always smaller than the other algorithms. The average normalized localization error of the DV-Hop algorithm is 0.3452, the average localization error of the PSODV-Hop algorithm is 0.2469, the average normalized localization error of the ABCDV-Hop algorithm is 0.2461, the average normalized localization error of the FADV-Hop algorithm is 0.2484, the average normalized localization error of the ISSADV-Hop algorithm is 0.1715, and the average normalized localization error of the HADSS algorithm is 0.1489. The analysis shows that the average normalized localization error of the HADSS algorithm is 56.87% smaller than that of the DV-Hop algorithm, 39.69% smaller than that of the PSODV-Hop algorithm, 39.50% smaller than that of the ABCDV-Hop algorithm, 40.06% smaller than that of the FADV-Hop algorithm, and 13.18% smaller than that of the ISSADV-Hop algorithm.

### 5.4. Effects of Anchor Node Numbers

**Figure 11:**
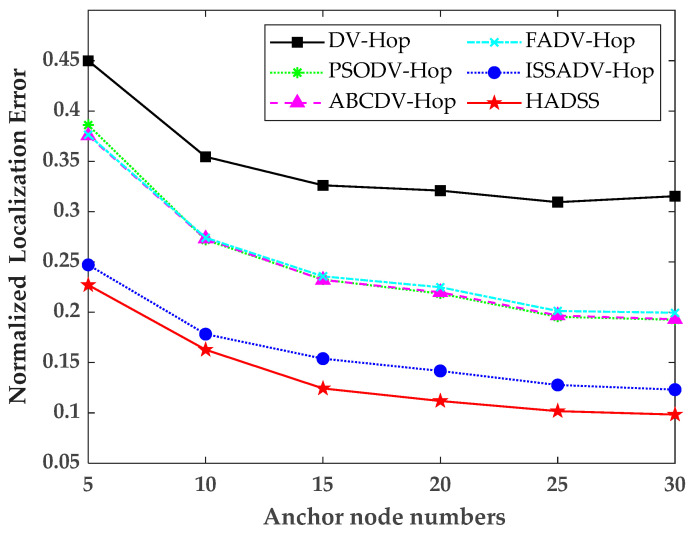
Variation in the normalized localization error for different anchor node numbers.

To study the localization performance of the HADSS algorithm with different anchor node numbers, the anchor node numbers were increased from 5 to 30, each time by 5, while keeping the communication radius, the sensor numbers, and the scenario sizes constant. The simulation results are shown in Figure 11 and Table 4.

From Figure 11 and Table 4, it can be concluded that with increasing anchor node numbers, the normalized localization error of all six algorithms tends to decrease, and the normalized localization error of the HADSS algorithm is always smaller than the other algorithms. The average normalized localization error of the DV-Hop algorithm is 0.3460, the average localization error of the PSODV-Hop algorithm is 0.2495, the average normalized localization error of the ABCDV-Hop algorithm is 0.2484, the average normalized localization error of the FADV-Hop algorithm is 0.2520, the average normalized localization error of the ISSADV-Hop algorithm is 0.1620, and the average normalized localization error of the HADSS algorithm is 0.1377. The analysis shows that the average normalized localization error of the HADSS algorithm is 60.20% smaller than that of the DV-Hop algorithm, 44.81% smaller than that of the PSODV-Hop algorithm, 44.57% smaller than that of the ABCDV-Hop algorithm, 45.36% smaller than that of the FADV-Hop algorithm, and 15.00% smaller than that of the ISSADV-Hop algorithm.

### 5.5. Effects of Scenario Sizes

**Figure 12:**
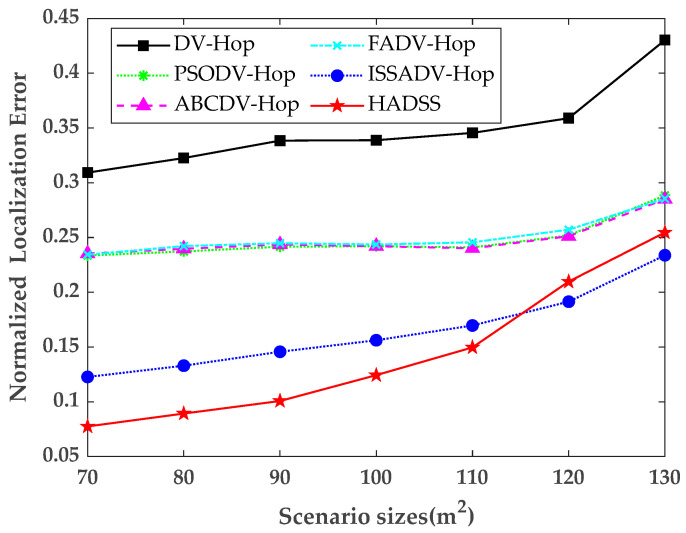
Variation in the normalized localization error for different scenario sizes.

To study the localization performance of the HADSS algorithm under different scenario sizes, the scenario sizes were increased from 70×70 m to 130×130 m, and the scenario edge length was increased by 10 m each time while keeping the communication radius, the anchor node numbers, and the sensor numbers constant. The simulation results are shown in Figure 12 and Table 5.

From Figure 12 and Table 5, it can be concluded that the normalized localization error of all six algorithms tends to increase as the scenario size continues to expand. The normalized localization error of the HADSS algorithm is always smaller than the other algorithms when the scenario size is smaller, and when the scenario size is larger than 110×110 m, the normalized localization error of the ISSADV-Hop algorithm is slightly smaller than that of the HADSS algorithm. The average normalized localization error of the DV-Hop algorithm is 0.3491, the average localization error of the PSODV-Hop algorithm is 0.2480, the average normalized localization error of the ABCDV-Hop algorithm is 0.2482, the average normalized localization error of the FADV-Hop algorithm is 0.2505, the average normalized localization error of the ISSADV-Hop algorithm is 0.1646, and the average normalized localization error of the HADSS algorithm is 0.1437. The analysis shows that the average normalized localization error of the HADSS algorithm is 58.84% smaller than that of the DV-Hop algorithm, 42.06% smaller than that of the PSODV-Hop algorithm, 42.10% smaller than that of the ABCDV-Hop algorithm, 42.63% smaller than that of the FADV-Hop algorithm, and 12.70% smaller than that of the ISSADV-Hop algorithm.

## 6. Conclusions

In this paper, the HADSS algorithm is proposed to improve the DV-Hop algorithm for the problem of large positioning error. First, an adaptive hop count strategy is proposed to reduce the error of the DV-Hop algorithm in calculating the minimum hop count between nodes, and a hop count correction factor is introduced to correct the minimum hop count twice, which refines the hop count and reduces the communication overhead compared with the multiple communication radii method. Second, the mean square error criterion is introduced to calculate the average hop distance between anchor nodes. A combination of the anchor node trust degree and the weighting methods is used to obtain the average hop distance, reducing the average hop distance error. Finally, the improved sparrow search mechanism is utilized to calculate the coordinates of the unknown nodes, by which the error of the least squares method in calculating the coordinates of the nodes is avoided. In ISSA, in order to enrich the diversity of the initial sparrow population, a good point set strategy is used to initialize the sparrow population. Then, the position-updating mechanism of discoverers in the sparrow population is improved so that the discoverers can perform a broader global search at the beginning of the population iteration and a more accurate local search at the end of the iteration. Finally, in order to prevent the sparrow population from falling into a local optimum, an adaptive t-distribution strategy is used to perturb the updated positions, which improves the algorithm’s search capability. The simulation experiments show that the HADSS algorithm has higher localization performance compared with the DV-Hop algorithm, PSODV-Hop algorithm, ABCDV-Hop algorithm, FADV-Hop algorithm, and ISSADV-Hop algorithm and effectively reduces the localization error in the original DV-Hop algorithm.

## Figures and Tables

**Table 1:** Simulation parameters settings.

Simulation Parameters	Value
Scenario sizes	100 × 100 m
Communication radius	30 m
Sensor numbers	100
Anchor node numbers	15
Unknown node numbers	85
Proportion of discoverers	0.2
Proportion of scouts	0.2
Number of populations	30
Number of iterations	40
*ST*	0.6

**Table 2:** Normalized localization error for different communication radii.

Communication Radius (m)	20	25	30	35	40	45
DV-Hop	0.4767	0.3607	0.3265	0.3272	0.3082	0.3118
PSODV-Hop	0.3081	0.2540	0.2359	0.2362	0.2332	0.2448
ABCDV-Hop	0.3065	0.2521	0.2354	0.2388	0.2346	0.2463
FADV-Hop	0.3198	0.2548	0.2390	0.2390	0.2373	0.2443
ISSADV-Hop	0.2702	0.1886	0.1483	0.1343	0.1316	0.1235
HADSS	0.3323	0.2050	0.1185	0.09248	0.08280	0.07674

**Table 3:** Normalized localization error for different sensor numbers.

Sensor Numbers	50	60	70	80	90	100	110
DV-Hop	0.3843	0.3642	0.3443	0.3322	0.3292	0.3278	0.3346
PSODV-Hop	0.2675	0.2611	0.2429	0.2468	0.2456	0.2353	0.2293
ABCDV-Hop	0.2671	0.2627	0.2396	0.2433	0.2443	0.2352	0.2305
FADV-Hop	0.2677	0.2643	0.2419	0.2454	0.2443	0.2389	0.2365
ISSADV-Hop	0.2024	0.1902	0.1762	0.1697	0.1579	0.1578	0.1466
HADSS	0.1935	0.1759	0.1541	0.1456	0.1292	0.1246	0.1197

**Table 4:** Normalized localization error for different anchor node numbers.

Anchor Node Numbers	5	10	15	20	25	30
DV-Hop	0.4499	0.3545	0.3261	0.3209	0.3094	0.3153
PSODV-Hop	0.3860	0.2720	0.2325	0.2185	0.1955	0.1925
ABCDV-Hop	0.3754	0.2731	0.2321	0.2199	0.1968	0.1931
FADV-Hop	0.3764	0.2741	0.2357	0.2249	0.2013	0.1995
ISSADV-Hop	0.2471	0.1783	0.1539	0.1417	0.1277	0.1231
HADSS	0.2271	0.1628	0.1243	0.1119	0.1018	0.09842

**Table 5:** Normalized localization error for different scenario sizes.

Scenario Sizes	70 × 70	80 × 80	90 × 90	100 × 100	110 × 110	120 × 120	130 × 130
DV-Hop	0.3093	0.3225	0.3384	0.3388	0.3455	0.3590	0.4303
PSODV-Hop	0.2335	0.2373	0.2416	0.2420	0.2410	0.2519	0.2885
ABCDV-Hop	0.2353	0.2397	0.2436	0.2421	0.2401	0.2512	0.2851
FADV-Hop	0.2343	0.2422	0.2448	0.2436	0.2456	0.2572	0.2855
ISSADV-Hop	0.1227	0.1330	0.1457	0.1562	0.1697	0.1914	0.2338
HADSS	0.07749	0.08930	0.1008	0.1243	0.1497	0.2097	0.2546

## Data Availability

All data are included in this work. No additional data are available.
